# Visual Imagery and False Memory for Pictures: A Functional Magnetic Resonance Imaging Study in Healthy Participants

**DOI:** 10.1371/journal.pone.0169551

**Published:** 2017-01-03

**Authors:** Christian Stephan-Otto, Sara Siddi, Carl Senior, Daniel Muñoz-Samons, Susana Ochoa, Ana María Sánchez-Laforga, Gildas Brébion

**Affiliations:** 1 Parc Sanitari Sant Joan de Déu, Universitat de Barcelona, Sant Boi de Llobregat, Spain; 2 Centro de Investigación Biomédica en Red de Salud Mental (CIBERSAM), Madrid, Spain; 3 Section of Clinical Psychology, Department of Education, Psychology, and Philosophy, University of Cagliari, Cagliari, Italy; 4 Facultat de Medicina, Universitat de Barcelona, Barcelona, Spain; 5 School of Life & Health Sciences, Aston University, Birmingham, United Kingdom; 6 Hospital Sant Joan de Déu, Esplugues de Llobregat, Spain; University of Groningen, NETHERLANDS

## Abstract

**Background:**

Visual mental imagery might be critical in the ability to discriminate imagined from perceived pictures. Our aim was to investigate the neural bases of this specific type of reality-monitoring process in individuals with high visual imagery abilities.

**Methods:**

A reality-monitoring task was administered to twenty-six healthy participants using functional magnetic resonance imaging. During the encoding phase, 45 words designating common items, and 45 pictures of other common items, were presented in random order. During the recall phase, participants were required to remember whether a picture of the item had been presented, or only a word. Two subgroups of participants with a propensity for high vs. low visual imagery were contrasted.

**Results:**

Activation of the amygdala, left inferior occipital gyrus, insula, and precuneus were observed when high visual imagers encoded words later remembered as pictures. At the recall phase, these same participants activated the middle frontal gyrus and inferior and superior parietal lobes when erroneously remembering pictures.

**Conclusions:**

The formation of visual mental images might activate visual brain areas as well as structures involved in emotional processing. High visual imagers demonstrate increased activation of a fronto-parietal source-monitoring network that enables distinction between imagined and perceived pictures.

## Introduction

Source monitoring refers to the ability to remember the origin of information. One type of source-monitoring process, referred to as ‘reality-monitoring’, ensures that the memories for perceived events are distinguished from those for imagined events. Such distinction can normally be achieved by assessing the context of the information: perceived information contains more sensory details, while imagined information has details about the cognitive operations executed during its formation [1]. Visual mental imagery might be critical in this distinction [2].

Reality-monitoring failure appears to be involved in hallucinations [3] and can occur in any modality. More particularly, failure to discriminate between imagined and perceived pictures might be involved in visual hallucinations [4]. Visual imagery and visual perception might have common neural substrates in healthy people as well as in people with visual hallucinations. Several groups have reported that visual imagery activates the same cortical regions as those involved in visual perception. Daselaar et al. [5] and Oertel et al. [6] observed activation in the lateral occipital complex (LOC) during visual imagery and visual hallucinations respectively, and the same area was also activated during visual perception in Daselaar et al. [5]. The lateral occipital complex is notably important for object perception [7,8]. In their review of neuroimaging studies of visual imagery, Kosslyn and Thompson [9] reported that the primary visual cortex (striate and calcarine cortex) was involved in visual imagery. On the other hand, certain studies have observed brain activity specific to either visual imagery or visual perception. Daselaar et al. [5] reported that the right superior parietal lobe was specifically involved in visual imagery. Ganis and colleagues [10] observed activity in the parietal cortex (especially the superior parietal lobule and the precuneus) and the prefrontal cortex during visual imagery and visual perception tasks, but the occipital cortex (inferior and right superior occipital gyrus) was engaged only during perception. The frontal and parietal regions appear to be involved in numerous types of cognitive control processes [11,12]. Visual imagery might engage brain areas involved in visual perception as well as those involved in cognitive control.

The neural mechanisms underlying false memories for pictures have been investigated at both the production (visual imagery) and retrieval (reality-monitoring judgment) phases. Gonsalves et al. [13] used a visual imagery task in which participants were asked to generate visual images on the basis of presented words. Pictures were presented as well. Participants were subsequently required to distinguish the pictures they had really seen from those they had imagined. The authors reported that, when processing the words that were later erroneously recalled as pictures, the participants activated the precuneus, right inferior parietal cortex, and anterior cingulate. Kensinger and Schacter [14] used a similar paradigm and found different areas to be engaged in these errors: the fusiform and parahippocampal gyri. Other studies have focused on the brain areas involved in the retrieval processes, i.e., the decision as to whether a picture has actually been presented or only imagined. Okado and Stark [15] found increased activation of the anterior cingulate gyrus during the false memories of pictures, while the parahippocampal gyrus and the occipital lobe were activated during the correct remembering of presented pictures. Kensinger and Schacter [16] reported that the left middle frontal gyrus was involved in the misattribution of items to pictorial presentation.

Although many studies have explored the misattribution of imagined pictures to perception, no functional neuroimaging study, as far as we know, has investigated proneness to visual imagery in relation to these errors. Visual imagery proneness might be critical in the false remembering of non-presented pictures. However, most studies explicitly requested the participants to generate visual mental images of the items, and therefore spontaneous visual imagery was not assessed.

Our objective was to determine, in healthy individuals prone to visual imagery, the neural bases of the confusion between spontaneously imagined pictures and perceived pictures, a mechanism that could be crucial in the formation of visual hallucinations. Pictures of common items mixed with the mere verbal labels of others were presented to participants, who were subsequently required to remember whether they had seen a picture of the item or only its verbal label. Cortical activity associated with both the processing and the recall of the verbal labels erroneously remembered as pictures was examined. A subgroup of participants with high and another with low visual imagery abilities were identified on the basis of a new scale specifically designed for this study. Although standard questionnaires of visual imagery are available, we created a tool thought to assess the spontaneous production of visual mental imagery rather than its vividness. People with high visual imagery were expected to activate vision-related brain areas when processing these verbal labels, suggesting that they had made a mental image of the item. Such brain areas may include the lingual gyrus, the middle occipital gyrus, or the fusiform girus, which have all been found to be involved in the viewing of pictures [17]. Additional activation of non-visual brain areas would suggest that these areas are involved in the process of forming a visual mental image. In the recall phase, people with high visual imagery were expected to activate brain areas related to source- monitoring judgment. These brain areas might include the anterior cingulate gyrus and the left middle frontal gyrus, as suggested by the above-mentioned studies of false remembering of imagined pictures. Propensity to non-clinical hallucinations and delusions was also assessed in order to examine the specificity of the alleged associations between visual imagery proneness and brain activity. Indeed, clinical and non-clinical psychotic experiences have been associated with reality-monitoring failure, and they may also be associated with visual imagery [18,19]. We set out to determine whether the brain activity observed in participants with high visual imagery proneness was similarly observed in participants with high hallucination and/or delusion proneness.

## Methods

### Participants

Twenty-six healthy participants (10 female) with normal or corrected-to-normal vision were recruited from the general population by means of announcements: age: mean = 37.3, sd = 9.1; education level: mean = 5.9 (the scale used was: 1 = no studies; 2 = uncompleted primary studies; 3 = completed primary studies; 4 = high school uncompleted; 5 = high school completed; 6 = undergraduate studies; 7 = bachelor’s or master’s degree; 8 = doctorate); verbal IQ: mean = 24.2, sd = 5.0 (verbal IQ was assessed by means of the *Acentuación de Palabras* test; the equivalent standardized verbal IQ for 24.2 is 104 [20]). The inclusion criteria were age between 18 and 60 years, and fluency in Spanish. The exclusion criteria were neurological or mental illness, intellectual disability, head injury, alcohol or drug abuse in the past six months, and current severe physical disease, as well as the standard exclusion criteria for participation in fMRI procedures, namely claustrophobia and metallic implants including fitted pacemaker and cochlear implants. This study was approved by the Parc Sanitari Sant Joan de Déu ethics committee and all participants provided written informed consent before taking part in the study.

### Scales for visual imagery, hallucinations and delusion proneness

Proneness to hallucinations was assessed by means of a Spanish adaptation of the Launay-Slade Hallucination Scale (LSHS; [21]), a self-questionnaire which assesses non-clinical hallucinations within various modalities. Two additional items were mixed with the LSHS items, although not taken into account in the computation of the global LSHS score: ‘*I can easily identify animals or things in the clouds’*, and ‘*When I see spots (of painting*, *humidity…)*, *I can see faces*, *silhouettes or objects in them’*. These two items were first piloted in the general population to ensure that they led to a diversity of scores. Similar to the LSHS items, each of these new items had to be rated from 0 to 3 by the participants according to the frequency of the experience. The extended LSHS scale including the two new items was validated in a Spanish sample of 210 non-clinical individuals, and the two added items demonstrated a satisfactory internal consistency (Cronbach’s *alpha* = .82; Sara Siddi, personal communication). The total score obtained on these two items constituted a visual imagery score (m = 1.77, sd = .95; range: 0–3) and was used to define two subgroups: nine participants who obtained a visual imagery score of 0 or 1 were included in the low visual imagery subgroup (mean score: m = .67), although one of them had to be subsequently ruled out of the encoding (but not of the recall) analyses due to unreliable fMRI data; and seven participants who scored at least 2 on either of the two questions were included in the high visual imagery subgroup (mean score: m = 2.86). The two subgroups were highly significantly different with regard to the visual imagery score (t(14) = 9.62, p <.0001). The remaining ten participants, who obtained a visual imagery score of 2 by scoring 1 on each of the two questions, were excluded from the analyses involving visual imagery score because it was unclear whether they should be included in the high or the low visual imagery subgroup.

A global hallucination proneness score was tallied by adding up the sub-scores for all LSHS items (excluding the two new items) (m = 4.5, sd = 3.2; range: 0–11). Subgroups of participants with high (score ≥ 6, n = 8 for encoding and n = 7 for recall) and low (score ≤ 2, n = 8 for encoding and n = 7 for recall) global hallucination proneness score were defined. Ten participants with a score of 3, 4, or 5 were excluded from the analyses contrasting these subgroups. Auditory non-verbal and verbal hallucination proneness scores were computed as well by adding up the sub-scores obtained on the corresponding items. Proneness to delusions was assessed by means of the Peters Delusion Inventory scale [22] (m = 4.7, sd = 4.5; range: 0–21). Subgroups of participants with high (score > = 6, n = 11 for encoding and n = 10 for recall) and low (score < = 2, n = 9 for encoding and for recall) delusion proneness score were defined. Five participants who scored 3, 4, or 5 were excluded from these subgroup comparisons. Certain fMRI data had to be discarded due to technical problems (see below).

### Material

Ninety items were selected, including 72 common objects (saw, apron, envelope…) and 18 vegetables (carrot, cauliflower, onion…). Participants were presented with either the mere verbal label of the item or the picture of the item along with its verbal label. Half of the items were presented as single words and the other half as word/picture pairs. Two versions of the stimuli were prepared; the 45 stimuli that were presented as words in one version were presented as word/picture pairs in the other. The use of each version was counterbalanced among subjects. All material was presented to participants throughout via Presentation (http://www.neurobs.com/). Before both the encoding and the recall phases, participants were administered a few practice trials to ensure familiarity with the task.

### Procedure

#### Encoding

All items were presented one by one in pseudo-random order with each slide (word or word/picture pair) being presented for 3.5 seconds, separated by fixation crosses. The stimulus onset asynchrony varied across trials from 5.5 to 9 seconds according to an exponential distribution with a mean of 6.68 seconds. This stimulus onset asynchrony distribution was used because a pilot study had indicated that it resulted in reliable activation patterns and was optimal in terms of total task duration. The encoding of the items was implicit since the task was described as a classification task: participants were required to indicate whether the presented items were vegetables or not, and to give their response by pressing one of two buttons (‘vegetable’ or ‘other’). They were not informed of the subsequent recall task. The computer recorded correct and incorrect responses, as well as misses (i.e., no response after 2 seconds from the end of the stimulus).

#### Recall

A 6-minute delay followed, during which a structural MRI scan was acquired for each participant. Then the participants were informed that they would be presented with all the labels of the stimuli they had previously seen, and that they would have to remember whether a picture accompanied the item at the encoding phase. The labels were presented one at a time for a 3.5-second period, in pseudo-random order different from that used in the encoding stage, but with similar stimulus onset asynchrony distribution. After the appearance of each label the participants were asked to provide their response by pressing one of two buttons (‘with picture’ or ‘without picture’). The correct responses, i.e., presented words remembered as words (WW) and presented pictures remembered as pictures (PP), were recorded, as were the incorrect responses: the omissions, i.e. presented pictures remembered as words (PW) and the false memories of pictures, i.e. presented words remembered as pictures (WP). Again, missed trials were recorded.

The response times for each type of response were recorded as well. Prior to the experimental protocol participants were provided with an opportunity to ask any questions they had.

### fMRI data acquisition

Functional MRI data were acquired with a 1.5T General Electric Signa HDe scanner at Parc Sanitari Sant Joan de Déu. A T2*-weighted functional echoplanar imaging sequence depicting BOLD contrast was obtained using a quadrature head coil. In total 270 volumes were collected with slices parallel to the AC-PC plane, resulting in an axial-oblique orientation. The following scanning parameters were used: 26 interleaved slices, 4 mm thickness, 1 mm gap, TR = 2000 ms, TE = 40 ms, 24 cm FOV, 64 × 64 acquisition matrix, flip angle = 90°. The first seven volumes in each run were discarded to allow for magnetic saturation effects. Visual stimuli were presented on a rear projection screen and viewed through a mirror mounted on the head coil, and all responses were collected with an MR-compatible response box (fORP, Current Designs, Inc., USA; www.curdes.com).

### fMRI data preprocessing

Imaging data were analyzed using SPM8 (Wellcome Department of Imaging Neuroscience, London; www.fil.ion.ucl.ac.uk/spm) running under MATLAB (Release 2009a, The MathWorks, Inc., Natick, Massachusetts). All of the volumes from each participant were spatially realigned to the first image in each series, in order to correct for small head movements and to generate a mean image for the functional series. Motion parameters were examined for each subject to ensure no movements larger than the voxel size were present (no runs were discarded). The resulting series were transformed into SRI24 space [23], with a standard EPI template as deformation target, and then spatially smoothed using a Gaussian kernel of 8 mm full-width-at-half-maximum.

Due to machine errors, data from the encoding task for one participant (from the low imagery subgroup) were disregarded, as were data from the recall task for two other participants (both among the ten participants excluded from the high- vs. low-imagery subgroups).

### fMRI data analysis

Preprocessed fMRI data were then analyzed with an event-related model, using SPM8. In order to assess random effects at the individual level, the activity associated with the experimental conditions was modeled with a hemodynamic response function (HRF). To avoid the inherent problems of currently available slice-timing correction techniques based on temporal realignment [24], we controlled slice timing latencies by including the time derivative of the HRF in the individual model as a nuisance parameter [25]. This method has proven effective in compensating for slice acquisition delays for TR = 2 s [25,26]. Displacement and rotation motion parameters were also included as confounds in the individual model. A 200 s high-pass filter cut-off was used to remove low frequency noise, together with an AR(1) model to correct for temporal autocorrelation.

For both tasks, four event types were determined by the responses in the recall task: WW, PP, PW, and WP. To compare each event, linear contrasts were constructed to test experimental effects of interest. These contrasts were entered into a second level analysis in which subjects were treated as a random effect. One-sample and two- sample t-tests were used to assess within-group and between-subgroup activations, respectively.

The resulting statistical parametric maps were generated with SPM8, initially using an uncorrected voxel-level threshold defined by T = 3.0 (p < 0.003) and a cluster extent threshold defined by a family-wise-error (FWE) corrected p < 0.05. In the event that the resulting activation clusters encompassed several brain structures, a more restrictive voxel-wise threshold, T = 3.8–4.0 (p < 0.0004), was applied to better identify local activation maxima in each brain structure, again followed by a p < 0.05 FWE-corrected threshold on the remaining clusters. Activation peaks were labeled according to the SRI24/TZO cortical parcellation map [23] based on the template described by Tzourio-Mazoyer et al. [27].

## Results

The number of presented pictures correctly remembered as pictures out of 45 presented pictures was m = 25.4 (sd = 7.4). The number of presented words erroneously remembered as pictures (false memories) out of 45 presented words was m = 9.5 (sd = 6.7). Longer response times were observed for the false remembering of non-presented pictures than for the correct remembering of presented pictures (m = 1860 ms, sd = 478 ms vs. m = 1576 ms, sd = 377 ms; t(25) = 4.43, p <.0001).

We identified neural correlates of the confusion between presented and imagined pictures with model-based fMRI. Four response types were identified for both the encoding and recall tasks, according to the responses recorded during recall: presented pictures remembered as pictures (PP), presented words remembered as words (WW), presented pictures remembered as words (PW), and presented words remembered as pictures (WP). Missed responses were not modeled since there were extremely few of them. Verbal IQ and sex were controlled for in the following fMRI analyses for their potentially confounding effects.

### Perception

The neural substrates involved in the perception of words and pictures in the encoding stage were highlighted with straightforward contrasts of *presented pictures (P) > presented words (W)*, and *presented words (W)> presented pictures (P)* (see Table 1, upper section). The presentation of word/picture pairs activated bilateral fusiform and lingual gyri, as well as the left middle occipital gyrus. The presentation of words without pictures activated the right supramarginal gyrus and the right inferior parietal lobe.

**Table 1:** Brain activation areas during perception and encoding in 25 participants, after controlling for sex and verbal IQ.

	Contrast	Cluster size and cluster-level significance (p_FWE_, p_FDR_)	Cluster peak coordinates*		Region
			SRI24	MNI	
**Perception**					
	P > W (voxel-level p_FWE_ < 0.05)	338 (**0.001, 0.001**)	**-27, -70, -16**	**-30, -73, -6**	Fusiform L
		639 (**0.001, 0.001**)	**24, -64, -13**	**28, -72, -6**	Fusiform R
		111 (**0.001, 0.001**)	**-3, -85, -19**	**-4, -89, -6**	Lingual R / L
		73 (**0.001, 0.001**)	**-33, -82, -4**	**-37, -82, 11**	Middle occipital gyrus L
	W > P	265 (**0.002**, **0.002**)	57, -49, 32	62, -40, 45	Supramarginal gyrus R and
			45, -52, 44	49, -41, 59	inferior parietal lobe R
**Encoding**					
Correct subsequent memories	PP > PW	203 (**0.006**, **0.006**)	-39, -64, -13	-43, -66, -4	Lateral occipital complex L
		101 (0.107, 0.055)	36, -64, -13	38, -66, -3	Lateral occipital complex R
	WW > WP	N. S.	-	-	-
	WW > PP	261 (**0.001**, **0.001**)	12, 23, 26	13, 32, 20	Anterior cingulate gyrus R
			-6, 23, 32	6, 33, 27	and med. sup. front. gyrus L
		264 (**0.001**, **0.001**)	3, -73, 26	-5, -67, 43	Precuneus R / L
		437 (**0.001**, **0.001**)	48, -61, 2	52, -59, 13	Middle temporal gyrus R,
			48, -70, 20	52, -65, 36	and angular R,
			39, -52, 35	42, -43, 49	and inferior parietal lobe R
		180 (**0.005**, **0.003**)	-63, -34, -7	-69, -34, -5	Middle temporal gyrus L
		91 (0.099, **0.050**)	-45, -67, 20	-50, -62, 35	Angular L
False subsequent memories of pictures	WP > WW	N. S.	-	-	-
	WP > PP	184 (**0.010**, **0.010**)	**57, -49, 29**	**63, -41, 41**	Supramarginal gyrus R and
			36, -67, 41	39, -57, 60	superior parietal lobe R

LOC = lateral occipital complex; SRI24 = SRI24 stereotactic coordinates; MNI = MNI stereotactic coordinates; p_FWE_ = family-wise error corrected p-value; p_FDR_ = false discovery rate corrected p-value.

* Peak-level corrected significance p_FWE_ <.05 marked in bold coordinates.

### Encoding

Characteristic brain activity for each trial type during the encoding phase is illustrated in Supporting Information (S1 Fig).

#### Correct subsequent memories

The contrast *presented pictures remembered as pictures (PP) > presented pictures remembered as words (PW)*, used to study the successful encoding of pictures, resulted in a bilateral activation of the lateral occipital complex. To study the successful encoding of word-only trials we used the contrast *presented words remembered as words (WW) > presented words remembered as pictures (WP)*, for which no significant activation was observed. However, when the contrast *presented words remembered as*

*words (WW) > presented pictures remembered as pictures (PP)* was considered, numerous activations were observed (see Table 1, middle section).

#### False subsequent memories of pictures

The contrasts *WP > WW* and *WP > PP* were investigated to study the brain activation associated with the encoding of words later erroneously remembered as pictures. The contrast *WP > PP*, used to evaluate whether false subsequent memories of non-presented pictures differed from correct subsequent memories of presented pictures, showed significant activation of the right supramarginal gyrus and right superior parietal lobe (see Table 1, lower section).

#### Role of visual imagery in encoding-related brain activation

The visual imagery score followed normal distribution. It correlated significantly with the auditory hallucination proneness score (r = .45, p <.025) and with the delusion proneness score (ρ = .45, p <.025), and therefore these two variables were controlled for in the following contrasts.

**Fig 1:**
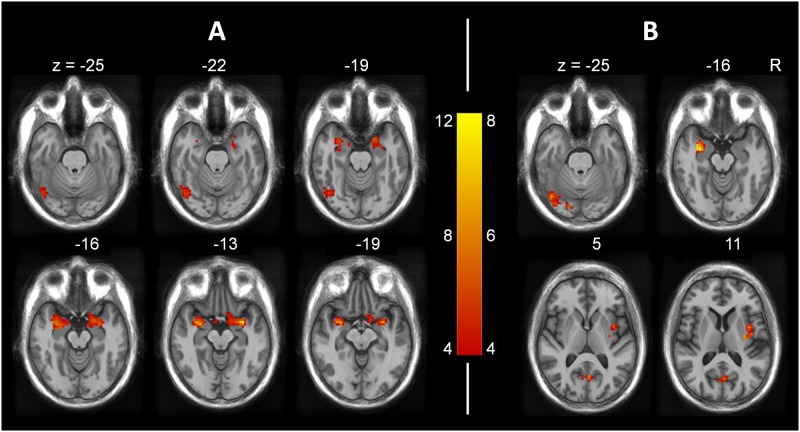
Activation maps consistent with false subsequent remembering of pictures in participants with high visual imagery.

To study the effect of visual imagery on the encoding of the items, we recomputed the above contrasts comparing the high (n = 7) and low (n = 8) visual imagery score subgroups by means of a two-sample t-test, after controlling for sex, verbal IQ, auditory hallucination proneness, and delusion proneness. Results are reported in Table 2. The contrasts for correct subsequent memories (PP > PW, WW > WP and WW > PP) did not yield any significant activation. However, the contrasts for false subsequent memories of pictures (WP > WW and WP > PP) yielded significant activation in various brain regions, notably the amygdala and the left inferior occipital gyrus (Fig 1).

**Table 2:** Brain activation differences between high (7 participants) and low (8 participants) visual imagery score subgroups during encoding, after controlling for verbal IQ, sex, auditory hallucination proneness, and delusion proneness.

	Contrast	Cluster size and cluster-level significance (p_FWE_, p_FDR_)	Cluster peak coordinates*		Region
			SRI24	MNI	
**Correct subsequent memories (encoding)**	PP > PW	N. S.	-	-	-
	WW > WP	N. S.	-	-	-
	WW > PP	N. S.	-	-	-
**False subsequent memories of pictures (encoding)**	WP > WW (T > 4.0)	114 (**0.002**, **0.012**)	**30, -1, -13**	**33, -1, -19**	Amygdala R
		94 (**0.006**, **0.018**)	-27, -4, -13	-29, -4, -19	Amygdala L
		60 (**0.046**, 0.086)	-39, -67, -22	-43, -71, -14	Inferior occipital gyrus L
	WP > PP (T > 4.0)	61 (**0.025**, **0.040**)	-30, -7, -16	-33, -8, -21	Amygdala L
		76 (**0.009**, **0.022**)	39, -10, 11	42, -5, 11	Insula R
		94 (**0.003**, **0.014**)	-39, -70, -25	-43, -75, -16	Inferior occipital gyrus L
		53 (**0.043**, 0.054)	3, -67, 5	3, -65, 18	Precuneus R / L

SRI24 = SRI24 stereotactic coordinates; MNI = MNI stereotactic coordinates;

p_FWE_ = family-wise error corrected p-value; p_FDR_ = false discovery rate corrected p-value.

* Peak-level corrected significance p_FWE_ <.05 marked in bold coordinates

To determine the specificity of this observed effect of visual imagery, we contrasted the subjects with high (n = 8) vs. low (n = 8) global hallucination proneness score after controlling for sex, verbal IQ, visual imagery score, and delusion proneness. The contrasts WP > WW and WP > PP did not yield any significant activation. Finally, contrasting the high (n = 11) vs. low (n = 9) delusion proneness scorers after controlling for sex, verbal IQ, visual imagery score, and auditory hallucination proneness score, did not yield any significant activation either.

### Recall

Characteristic brain activity for each trial type during the recall phase is illustrated in Supporting Information (S2 Fig).

Analyses were conducted on the recall data in 24 participants, to study brain activations associated with the remembering of whether a picture of the item was presented at the encoding phase (see Table 3).

**Table 3:** Brain activation areas during recall in 24 participants, after controlling for sex and verbal IQ.

	Contrast	Cluster size and cluster-level significance (p_FWE_, p_FDR_)	Cluster peak coordinates*		Region
			SRI24	MNI	
**Correct memories (recall)**	PP > PW (T = 3.90)	91 (**0.008**, **0.037**)	**-6, -88, -16**	**-7, -91, -1**	Calcarine L + Lingual L
		94 (**0.007**, **0.037**)	6, 11, 2	7, 14, -5	Caudate R
		89 (**0.009, 0.037**)	-9, 2, 8	-10, 6, 4	Caudate L
		57 (**0.038,** 0.094)	-6, -79, 23	-7, -73, 41	Precuneus L
		69 (**0.022,** 0.067)	-42, -67, 35	-46, -59, 52	Inferior parietal lobe L and
			-39, -67, 23	-43, -61, 38	angular gyrus L
	PP > WW (T = 4.00)	369 (**0.001**, **0.001**)	-15, -4, 23	-16, 3, 23	Caudate L and
			9, -4, 14	10, 2, 13	caudate R
		49 (**0.021**, 0.123)	-6, -88, -16	-7, -91, -1	Calcarine L + Lingual L
	WW > WP	N. S.	-	-	-
	WW > PP	N. S.	-	-	-
**False memories of pictures (recall)**	WP > WW	N. S.	-	-	-
	WP > PP	N. S.	-	-	-

SRI24 = SRI24 stereotactic coordinates; MNI = MNI stereotactic coordinates;

p_FWE_ = family-wise error corrected p-value; p_FDR_ = false discovery rate corrected p-value.

* Peak-level corrected significance p_FWE_ <.05 marked in bold coordinates.

#### Correct memories

The same analyses as those conducted in the encoding data showed activation only in the contrast *presented pictures remembered as pictures* > *presented pictures remembered as words* (PP > PW). In addition, we included the contrast *presented pictures remembered as pictures > presented words remembered as words* (PP > WW), which was not studied for the encoding phase since it was equivalent to P > W in terms of its effect of interest. This latter contrast also yielded significant activations.

#### False memories of pictures

No significant activation was observed for the contrasts reflecting the false memories of non-presented pictures.

#### Role of visual imagery in recall-related brain activation

**Fig 2:**
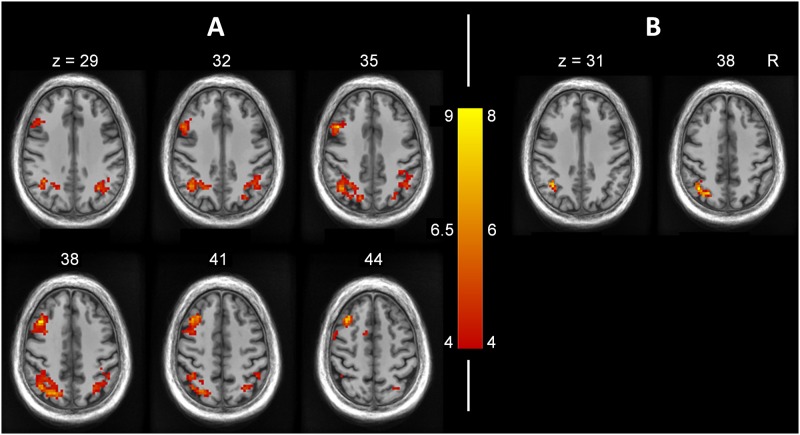
Activation maps consistent with false remembering of pictures in participants with high visual imagery. Brain activity differences between high- (n = 7) vs. low- (n = 9) visual imagery score subgroups when controlling for verbal IQ, sex, auditory hallucination proneness, and delusion proneness (see Table 4). Activations are shown over the SRI24 structural template for illustration purposes only. A) Activation of the left middle frontal gyrus and bilateral activation of the inferior and superior parietal lobes during the false remembering of non-presented pictures when compared to correct remembering of words presented as words (contrast WP > WW). B) Activation of the left inferior and superior parietal lobe during the false remembering of non-presented pictures when compared to correct remembering of presented pictures (contrast WP > PP).

These contrasts were recomputed comparing the high (n = 7) vs. low (n = 9) visual imagery scoring participants. Results are presented in Table 4. A significant activation of the left precentral and postcentral gyri was observed for the correct remembering of presented pictures (PP > WW). The false memories of pictures (WP > WW and WP > PP) led to activations in the left middle frontal gyrus and the inferior and superior parietal lobes (Fig 2).

**Table 4:** Brain activation differences between high (7 participants) and low (9 participants) visual imagery score subgroups during recall, after controlling for verbal IQ, sex, auditory hallucination proneness, and delusion proneness.

	Contrast	Cluster size and cluster-level significance (p_FWE_, p_FDR_)	Cluster peak coordinates*		Region
			SRI24	MNI	
**Correct Memories (recall)**	PP > PW	N. S.	-	-	-
	PP > WW (T > 3.8)	104 (**0.007**, **0.024**)	-30, -43, 50	-33, -31, 64	Precentral gyrus L and postcentral gyrus L
	WW > WP	N. S.	-	-	-
	WW > PP	N. S.	-	-	-
**False memories of pictures (recall)**	WP > WW (T > 4.0)	199 (**0.001**, **0.001**)	**-36, 17, 38**	**-39, 28, 35**	Middle frontal gyrus L
		197 (**0.001**, **0.001**)	-36, -64, 38	-40, -55, 55	Inf. parietal lobe L and
			-21, -70, 38	-23, -61, 56	sup. parietal lobe L
		141 (**0.001**, **0.001**)	30, -61, 29	32, -53, 44	Inf. parietal lobe R and
			27, -64, 41	29, -54, 59	sup. parietal lobe R
	WP > PP (T > 4.0)	55 (**0.036**, 0.081)	-36, -61, 38	-40, -52, 54	Inf. parietal lobe L and
			-24, -67, 38	-27, -58, 56	sup. parietal lobe L

SRI24 = SRI24 stereotactic coordinates; MNI = MNI stereotactic coordinates; p_FWE_ = family-wise error corrected p-value; p_FDR_ = false discovery rate corrected p-value.

* Peak-level corrected significance p_FWE_ <.05 marked in bold coordinates.

To determine the specificity of the effects of visual imagery, we contrasted the subjects with high (n = 7) vs. low (n = 7) global hallucination proneness score after controlling for sex, verbal IQ, visual imagery score, and delusion proneness score. No significant activations were observed for the false memories related contrasts WP > WW and WP > PP. Contrasting the high (n = 10) vs. low (n = 9) delusion proneness scorers after controlling for sex, verbal IQ, visual imagery score, and auditory hallucination proneness score did not yield any significant activation in those contrasts either.

#### Associations of visual imagery with scores and response times in the recall task

Finally, we conducted regression analyses on the scores and response times for each type of response within the 16 participants from the high and low visual imagery subgroups. With respect to the scores, subgroups (high/low), auditory hallucination proneness score, delusion proneness score, sex, and verbal IQ were used as predictors. No significant or trend level association with visual imagery was observed for any of the four scores (number of pictures correctly remembered as pictures, number of pictures remembered as words, number of words remembered as pictures, and number of words correctly remembered as words). With respect to the response times, the same predictors were used except that sex was replaced by age. Visual imagery subgroup membership was significantly associated with the response time for the false memories of non-presented pictures (see Table 5). Data showed that the 7 participants with high visual imagery score presented longer response times for these false memories than did the 9 participants with low visual imagery score (estimated marginal means: 2224 ms vs. 1596 ms).

**Table 5:** Regression analyses of the response time for each type of response in the recall task, in the 16 participants with high (n = 7) and low (n = 9) visual imagery scores (β coefficients and p values).

Type of response	High vs low visual imagery scorers	Auditory hallucination proneness	Delusion proneness	Age	Verbal IQ	R2	F test	P value
Correct memories of presented pictures (PP)	.52	-.55	-.25	-.12	-.56	.52	2.2	ns
Omission of presented pictures (PW)	.09	-.48*	.13	.53*	-.26	.82	9.0	.002
False memories of pictures (WP)	.83**	-.35	-.37	-.25	**-.77**+	.64	3.5	.05
Correct memories of presented words (WW)	.07	-.41	-.16	.50	-.18	.74	5.8	.009

^+^ p = .051,

* p <.05,

** p <.01.

## Discussion

The purpose of this study was to investigate the neurophysiological underpinnings of the formation and subsequent false remembering of imagined pictures in individuals with high visual imagery abilities. It was expected that the presentation of words designating common items would facilitate the formation of visual mental images, and therefore the participants would have difficulty discriminating afterwards whether the picture was really perceived before, or only imagined.

As expected, when processing the words later remembered as pictures, people prone to visual imagery activated a visual area, namely the left inferior occipital gyrus [28], significantly more than did the other participants. It should be noted that, in the whole sample, the left middle occipital gyrus was involved in the perception of pictures, and the lateral occipital complex was involved in the effective encoding of these pictures (see Table 1). Activation in visual areas has been associated with the formation of visual mental images in a number of investigations [5,6,8]. In their whole sample, Ganis et al. [10] found the inferior occipital area to be implicated during perception of pictures but not during visual imagery. Taken together, their results and ours suggest that only people with high visual imagery abilities activate the occipital gyrus when presented with words designating common objects.

When processing words later erroneously remembered as pictures, people with high visual imagery abilities also activated the amygdala, the insula and the precuneus significantly more than did their counterparts with lower imaging abilities. The amygdala and insula have been found to be involved in emotional processing [29–31]. The insula has also been seen to be involved in self-reflective processes [32] as well as in visual imagery and perception [10]. As for the amygdala, it has also been implicated in consolidation and retrieval processes [33]. Ford et al. [34] demonstrated hyperconnectivity between the amygdala and visual cortex in schizophrenia patients with visual hallucinations, and they argued that this hyperconnectivity may facilitate activation of emotional visual images. With respect to the precuneus, it has been found to be implicated in various functions such as visual processing, visuo-spatial imagery, episodic memory retrieval, and self-processing operations [35,36]. Gonsalves et al. [13] reported, as did we, that the precuneus was activated when processing words later remembered as pictures. The activation of these brain areas, namely amygdala, insula, and precuneus, along with the occipital gyrus, might be responsible for the formation of imagined pictures.

In the subsequent source memory judgment, those individuals with high visual imagery abilities, when erroneously remembering pictures, showed greater activation in the left middle frontal gyrus than did the other participants, as well as greater bilateral activation of the inferior and superior parietal lobe. Kim and Cabeza [37] observed that high confidence false recognition of words was similarly associated with fronto-parietal activity, and they interpreted this activity as reflecting the processing of familiarity. The parietal lobe is associated with declarative memory [38] and source memory [39]. More particularly, it has been associated with false memories and retrieval of contextual information [15,40–42] as well as with decision making [43]. As for the middle frontal gyrus, it is generally involved in executive functions [44] and spatial working memory [45]. In agreement with our data, previous studies found the middle frontal gyrus to be involved in pictorial misattributions [16] and false recognition of new words [46]. On retrieval, participants with high visual imagery might attempt to remember the sensory details associated with the imagined pictures in order to make their decisions. This effort was also reflected in their increased response time when falsely remembering pictures, relative to the participants with low visual imagery ability. However, high visual imagery abilities were not associated with increased rates of false memories of pictures. Thus, in these non-clinical individuals with presumably intact reality-monitoring skills, the visual mental images formed during word presentation seem to have led mostly to enhanced recruitment of reality-monitoring resources on memory test in order to resolve the conflict between imagined and perceived pictures. It is important to note that the effects observed are specific to individuals prone to visual imagery, since no differential brain activity was observed in individuals prone to either hallucinations or delusions.

The main limitation of our study is that only small subgroups of participants were involved in the comparisons of high- vs. low-imagers, thereby reducing the reliability of the fMRI analyses. A sample of seven individuals with high visual imagery abilities is clearly insufficient to allow conclusions to be drawn on the role of visual imagery in reality monitoring processes. Moreover, due to the constraints of the fMRI procedure, the main measure of interest–false memories of pictures–relied on a limited number of observations. Further studies are definitively warranted to confirm the effects observed in our data. Indeed, we cannot rule out the possibility that these effects emerged merely by chance. Alternatively, though, the fact that significant differential brain activity between high- and low-imagers was recorded during both encoding and recall phases under these experimental conditions might mean that the effects are robust enough to break through in spite of limited power.

Another important limitation is that the propensity to visual imagery, which is central to the hypotheses, was assessed by means of a novel scale with a restrictive range. It would have been of interest to use a standard instrument such as the Vividness of Visual Imagery Questionnaire [47] along with our own and then compare the performance of both. However, what we sought to capture in this study was the spontaneity and abundance of visual imagery rather than its vividness, and our visual imagery scale appears to have been effective enough for that purpose. Indeed, it enabled us to identify a subgroup of individuals with high propensity to generating visual mental images as reflected by their differential activation of a visual area during word encoding. It should be noted, nonetheless, that our sample as a whole presented lower than average propensity to visual imagery, as demonstrated retrospectively by a validation study of the two items designed to investigate this dimension (visual score: m = 1.77, range: 0–3 in our whole sample vs. m = 2.33, range: 0–6 in 210 participants from the general population; Sara Siddi, personal communication). More striking results would probably have emerged if participants more strongly inclined to visual imagery had been included. An additional limitation is that our visual imagery score only reflected subjective ratings of participants’ visual imagery abilities. Different results might have been obtained with an objective measure of visual imagery.

Considering the small size of the sample and the use of a single restricted measure of visual imagery, our findings must be considered as preliminary. The results need to be replicated in larger samples, and using a more extensive assessment of objective and subjective visual imagery. In spite of these limitations, our data suggest that brain regions allegedly involved in emotional, self-reflective, and retrieval processes participate in the formation of visual mental images, along with visual areas. Furthermore, the left middle frontal gyrus and the parietal lobes seem to be involved in the reality-monitoring process that allows those visual mental images to be distinguished from perception. The study of patients with clinical hallucinations, such as schizophrenia and Parkinson’s disease patients, would enable us to investigate whether the same brain structures are involved in visual hallucinations. Further, analogous procedures involving verbal, rather than visual, material would enable us to determine whether the confusion between inner speech and perceived voices in patients with verbal hallucinations is similarly subserved by a fronto-parietal cortical network.

## Supporting Information

S1 Fig Brain activity for each trial type during the encoding phase. Common brain activity observed in 25 participants during encoding, after controlling for sex and verbal IQ. Rows illustrate the four trial types: presented words later remembered as words (WW), presented words later remembered as pictures (WP), presented pictures later remembered as pictures (PP), and presented pictures later remembered as words (PW). A FWE-corrected voxel-level p < 0.05 was applied.
(TIF)

S2 Fig Brain activity for each trial type during the recall phase. Common brain activity observed in 24 participants during recall, after controlling for sex and verbal IQ. Rows illustrate the four trial types: presented words remembered as words (WW), presented words remembered as pictures (WP), presented pictures remembered as pictures (PP), and presented pictures remembered as words (PW). A FWE-corrected cluster-level p < 0.05 was applied after an uncorrected voxel-level p < 0.0003 (T = 4).
(TIF)
