# Supplementary figures and images for: Visual Imagery and False Memory for Pictures: A Functional Magnetic Resonance Imaging Study in Healthy Participants
Source: PLoS One. 2017 Jan 3;12(1):e0169551. doi: 10.1371/journal.pone.0169551 (PMC5207728; doi:10.1371/journal.pone.0169551)

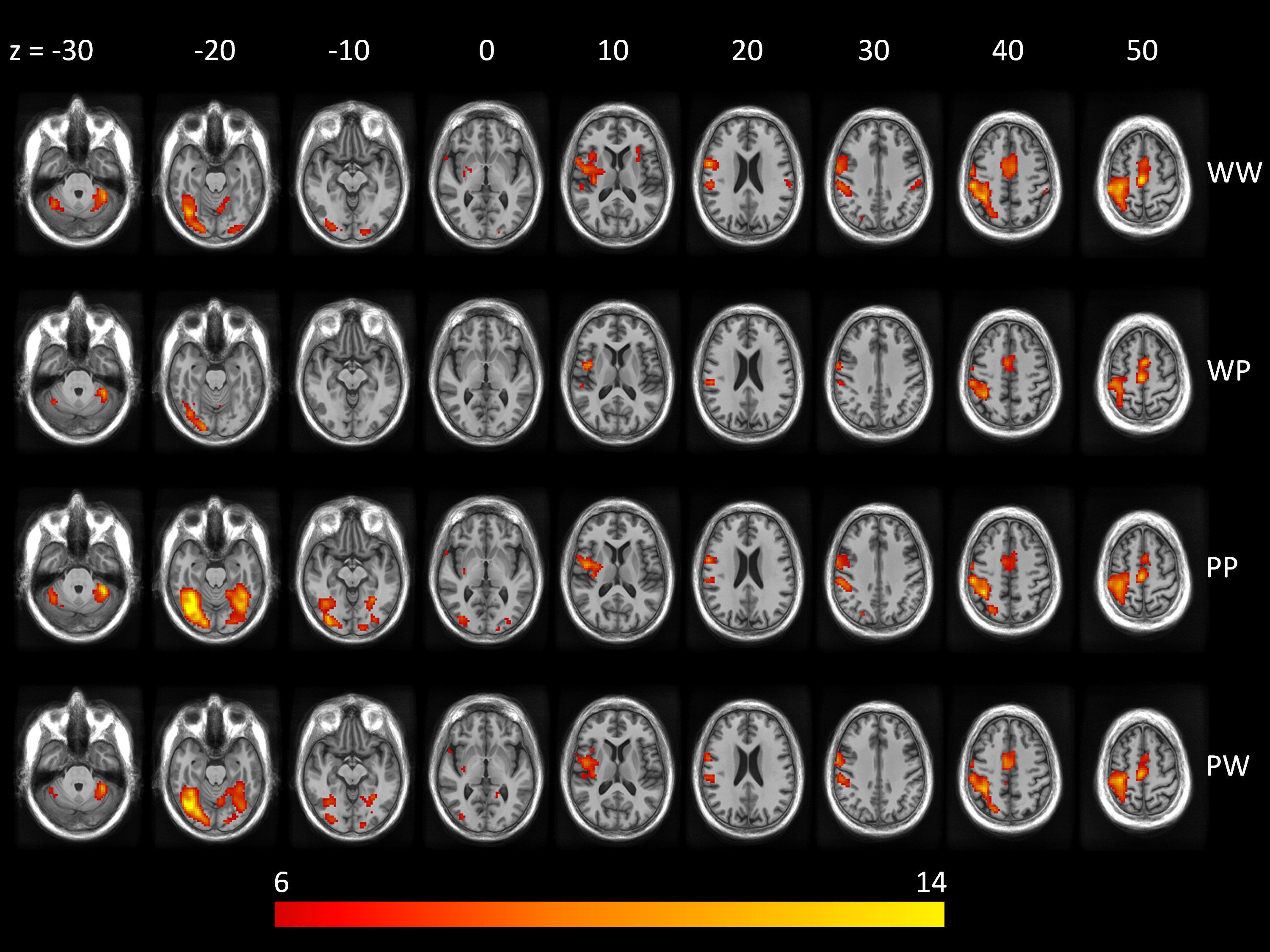

Supplement: S1 Fig — Common brain activity observed in 25 participants during encoding, after controlling for sex and verbal IQ. Rows illustrate the four trial types: presented words later remembered as words (WW), presented words later remembered as pictures (WP), presented pictures later remembered as pictures (PP), and presented pictures later remembered as words (PW). A FWE-corrected voxel-level p < 0.05 was applied. (TIF) [file pone.0169551.s001.tif]

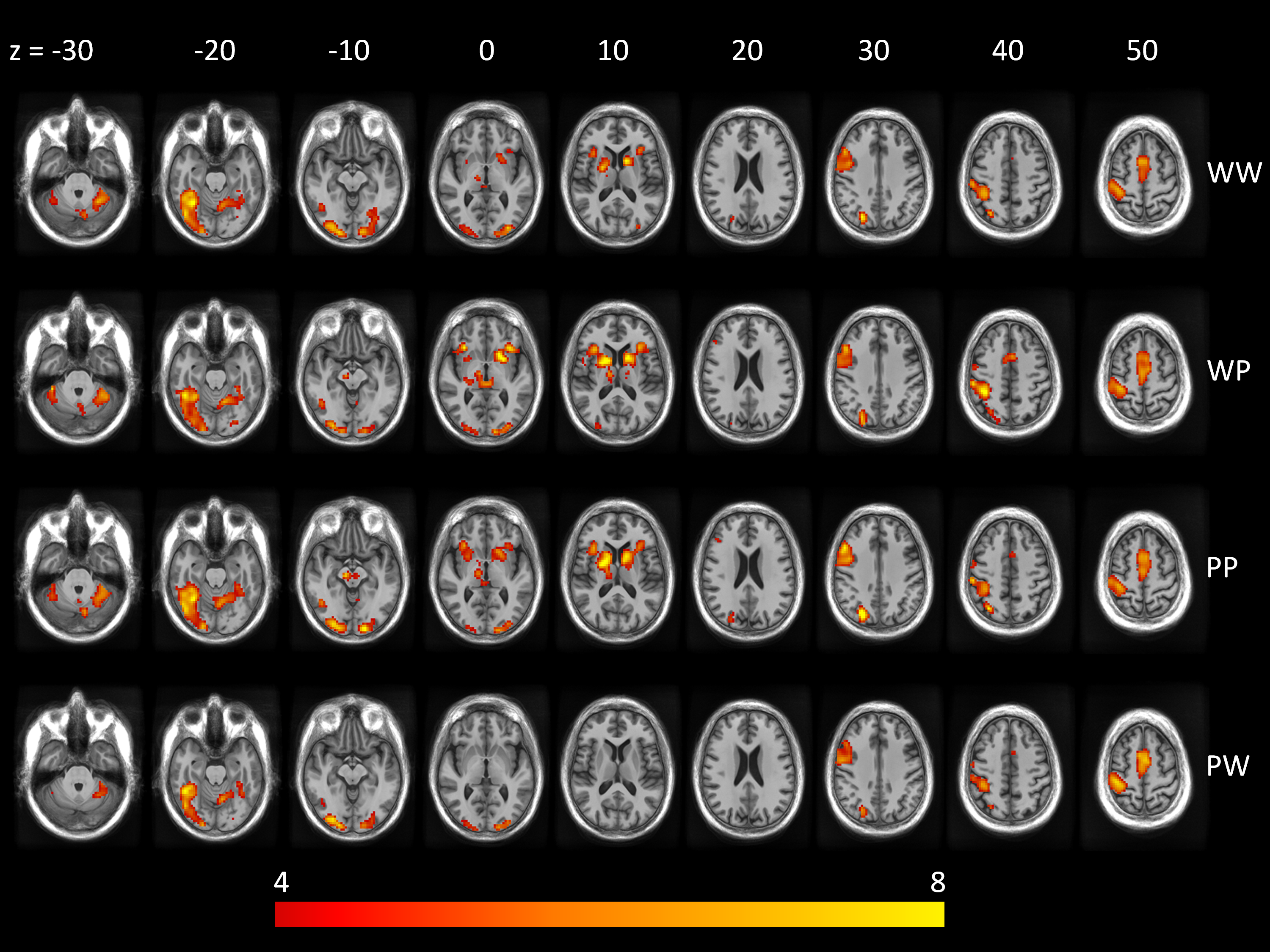

Supplement: S2 Fig — Common brain activity observed in 24 participants during recall, after controlling for sex and verbal IQ. Rows illustrate the four trial types: presented words remembered as words (WW), presented words remembered as pictures (WP), presented pictures remembered as pictures (PP), and presented pictures remembered as words (PW). A FWE-corrected cluster-level p < 0.05 was applied after an uncorrected voxel-level p < 0.0003 (T = 4). (TIF) [file pone.0169551.s002.tif]
